# Development of oral health policy in Nigeria: an analysis of the role of context, actors and policy process

**DOI:** 10.1186/s12903-015-0040-8

**Published:** 2015-05-06

**Authors:** Enyi Etiaba, Nkoli Uguru, Bassey Ebenso, Giuliano Russo, Nkoli Ezumah, Benjamin Uzochukwu, Obinna Onwujekwe

**Affiliations:** Department of Health Administration and Management, College of Medicine, University of Nigeria, Enugu Campus, Enugu, Nigeria; Department of Pharmacology and Therapeutics, Health Policy Research Group, College of Medicine, University of Nigeria, Enugu Campus, Enugu, Nigeria; Nuffield Centre for International Health and Development, Leeds Institute for Health Sciences, University of Leeds, Leeds, LS2 9JT UK; Instituto de Higiene e Medicina Tropical (IHMT), The Nova University of Lisbon, Campus de Campolide, 1099-085 Lisbon, Portugal; Department of Preventive Dentistry, College of Medicine, University of Nigeria, Enugu Campus, Enugu, Nigeria; Department of Sociology/Anthropology, University of Nigeria, Nsukka, Nigeria; Department of Community Medicine, College of Medicine, University of Nigeria, Enugu Campus, Enugu, Nigeria

**Keywords:** Oral health policy, Process, Context, Actors, Oral health

## Abstract

**Background:**

In Nigeria, there is a high burden of oral health diseases, poor coordination of health services and human resources for delivery of oral health services. Previous attempts to develop an Oral Health Policy (OHP) to decrease the oral disease burden failed. However, a policy was eventually developed in November 2012. This paper explores the role of contextual factors, actors and the policy process in the development of the OHP and possible reasons why the current approved OHP succeeded.

**Methods:**

The study was undertaken across Nigeria; information gathered through document reviews and in-depth interviews with five groups of purposively selected respondents. Analysis of the policy development process was guided by the policy triangle framework, examining context, policy process and actors involved in the policy development.

**Results:**

The foremost enabling factor was the yearning among policy actors for a policy, having had four failed attempts. Other factors were the presence of a democratically elected government, a framework for health sector reform instituted by the Federal Ministry of Health (FMOH). The approved OHP went through all stages required for policy development unlike the previous attempts. Three groups of actors played crucial roles in the process, namely academics/researchers, development partners and policy makers. They either had decision making powers or influenced policy through funding or technical ability to generate credible research evidence, all sharing a common interest in developing the OHP. Although evidence was used to inform the development of the policy, the complex interactions between the context and actors facilitated its approval.

**Conclusions:**

The OHP development succeeded through a complex inter-relationship of context, process and actors, clearly illustrating that none of these factors could have, in isolation, catalyzed the policy development. Availability of evidence is necessary but not sufficient for developing policies in this area. Wider socio-political contexts in which actors develop policy can facilitate and/or constrain actors’ roles and interests as well as policy process. These must be taken into consideration at stages of policy development in order to produce policies that will strengthen the health system, especially in low and middle-income countries, where policy processes and influences can be often less than transparent.

## Background

Whilst most developed countries of the world have oral health policies that are targeted towards oral disease prevention [1], a major barrier to improving oral health in the African Region is the absence of oral health policies to guide oral health activities [2]. Some of the factors that can influence health policy environment in low and middle income countries (LMICs) include the kind of health system operated by the country, their purchasing power, the influence of the private sector and the level of international influence on the health system [3]. A comparative policy analysis of four pairs of LMICs (Bangladesh/Pakistan, Thailand/Philippines, Tunisia/Algeria and Zimbabwe/Zambia) conducted to understand why some countries develop appropriate and effective programmes while other countries do not, identified that institutional and financial stability were amongst other factors that supported or inhibited the adoption of strong population policies and family planning programmes [4]. The ‘combination of different systemic factors (political, economic, social or cultural, both national and international) which may have an effect on health policy’ is often referred to as the *‘context’* [5,6], and this includes the environment within which institutions operate. The Overseas Development Institute considers internal context as separate from external influences (socio-economic and cultural issues and donor policies), that shape the relations between policy actors and the uptake (or not) of evidence in policy development [7]. Conversely, Dobrow *et al.* regard internal context more precisely as the environment in which a policy decision is *made* (guided by purpose of policy, actors’ participation and process of decision-making) and the external context as the environment in which a decision is *applied* including the wider political, disease-specific and extra-jurisdictional influences on policy implementation [8]. The foregoing suggests that exploring the influence of context on policies constitutes an important component of health policy analysis. Better understanding of the complex interactions between context, policy process and actors can help policymakers design more responsive and effective policies [9].

Prior to September 1998, approaches to oral health in most African countries consisted largely of the provision of unplanned, ad hoc and spasmodic curative dental services [10]. To improve this situation, the World Health Organization (WHO) Regional Committee for Africa, adopted a Regional Oral Health Strategy for the period 1999-2008 that aimed to: i) strengthen the capacity of member countries to improve community oral health; and ii) develop appropriate national oral health policies and implementation plans with emphasis on prevention, early detection and management of oral diseases [10]. The experiences of some African countries at various times in approaching an oral health policy show that contextual factors played a significant role in policy development process [11-13]. Other factors which influenced policy development included: i) use of evidence and the characteristics of evidence used in policymaking; ii) the policy process utilized and the role and relationship of actors; and iii) the ideologies of policy actors and ideologies of those who the policy is targeted at [1,3,14]. However there is still a dearth of empirical reports of specific and comparative analysis of the OHP policy processes in the African Region.

All through the various attempts at health reforms in the Nigerian health system, the delivery of oral health services had historically not been guided by any policy. However, at the latest health reform namely the National Strategic Health Development Plan (NSHDP) in 2010, all health areas were required to produce a policy to guide the development of the strategic plan document [15]. This and other factors highlighted above thus provided the impetus for developing a national oral health policy.

Policy making in Nigeria is usually a very slow process involving a number of stages during which key issues are debated and negotiated and evidence in support of the policy is examined before the policy is adopted as official government policy. Following this process, it can take a few years before a proposed policy is finally implemented and its impact felt. Most policies in Nigeria are deliberate choices, based on political mechanism, government oversight and usually lack appropriate information i.e. weak research-to-policy linkages [16]. Despite having one of the largest numbers of policy research institutions and think tanks in Africa, these are generally weak and unreliable [17]. This has partly been attributed to many years of military rule, bad governance, and a high level of corruption, especially during the period of military rule between the late 1980s and early 1990s, when most research establishments suffered from low funding, decay of infrastructure, and a flight of highly qualified academics to western institutions [18]. As the military régimes ruled largely by enacting decrees rather than through consultative policy development processes, health and health policies were not given priority. However, the return to civil rule in 1999 ushered in a stronger role for research institutions in policymaking by facilitating the inclusion of academics and policy experts in the policymaking process. Similarly, from 1999 onwards, an increasing number of technocrats, policy entrepreneurs and advocates of evidence-informed policies have been incorporated into the cabinet [19].

Nigeria operates a three-tier health system (i.e. federal, state and local government levels) but all health policies are usually made at the federal (national) level though some are adapted to each state’s context. Following policy development, the Federal Executive Council (FEC) is responsible for approving all policies before they can be adopted. The FEC is made up of the President of Nigeria and his Ministers, with the President as the chairman. This body initiates the policies and programmes of the Federal Government and ensures that they are properly implemented after they are passed into law by the legislature.

In Nigeria, most of the research evidence in support of oral health have been sporadic and based on convenience sample studies. This was attributed to lack of funds required to carry out national studies given the size of the country [20]. However, a national study conducted in 2004 showed a high burden of oral diseases with prevalence between 8-15% for various oral conditions [21]. Other studies in 1990s and 2000s identified four notable trends: i) a socioeconomic and geographic variation with an urban vs. rural disparity in oral disease prevalence and a higher disease burden in the northern part Nigeria [21] ii) an inequitable distribution of oral health facilities with the majority of facilities situated in the southern part of the country and in urban areas [22]; iii) uncoordinated national strategy for preventive dental services [20]; and iv) similar to most African countries, there is a poor health expenditure on oral health in Nigeria as the limited resources are directed towards life-threatening conditions like HIV/AIDS [23]. In addition, a detailed appraisal of the oral health care system in Nigeria, which analyses strengths, weaknesses, opportunities and threats (SWOT) clearly identified as a threat, the absence of clear guidelines and strategies to address oral health issues nationally [24].

To bolster the national oral health system, the oral health policy (OHP) in Nigeria was developed and finally adopted in November 2012 through multi-stakeholder participation of experts in oral health, WHO, and medical practitioners in the three tiers of the health system [16]. The OHP is intended to achieve optimal oral health for at least 50% of Nigerians through 5 strategies namely: i) sustainable awareness creation, ii) early detection and prompt treatment of oral diseases using evidence-based interventions, iii) strategic research, iv) workforce development; and v) coordination of oral health activities including institutionalization of modern dental practices [25].

Following recent calls to strengthen the field of health policy analysis and policy processes in LMICs [26], policy analysts have employed a wide range of tools in form of theories, models and frameworks to explain the processes and other components of policy making which remain complex [3,27,28]. For example, scholars have responded to calls for strengthening the field of policy analysis by illuminating processes of policy development for mental health and health financing in groups of African countries [29,30]. To our knowledge, there are neither empirical reports of comparative analysis of OHP policy processes in groups of LMICs nor reports of OHP policy processes in specific African countries. This paper provides new evidence on the policy process and the roles of context and policy actors in the development and approval of OHP in Nigeria. It hence provides knowledge and insight that can enhance future policy development and policy implementation especially for perceived neglected diseases in Nigeria and other developing countries.

## Methods

The study was undertaken in Nigeria, a West African country with a 2014 population of about 177 million people [31]. Nigeria is divided into six geo-political zones and 36 states in addition to a Federal Capital Territory. The country is a Federal Republic, with the 36 states acting as the federating units. The country operates a presidential system of democratic government and the federal government is led by an elected president. Each state government is led by an elected state governor.

To understand the the roles of actors, process and context in the development of the OHP, a case study approach was adopted [32], to facilitate exploration of the complex phenomenon of policy-making within the real-life context [33]. An important strength of the case study approach is that it calls for the use of multiple sources of data to inform analysis thereby: i) enhancing the potential for generating comprehensive and multi-faceted accounts of the case, and ii) reducing the chance that interpretation of the data will be misleading [34].

Two data collection methods were used: document review and in-depth interviews. Document review was used at the initial stage of the research to identify the different activities surrounding the development of the OHP and inform the development of the initial list of respondents for the study. The documents reviewed for this research included: the new OHP; previous National Oral Health Policies for Nigeria 2009-2013 (that were not Approved); the National Strategic Health Development Plan (NSHDP); the National Health Bill; the National Health Policy and the Nigerian National Constitution. Information was extracted in a standardized format and coded according to information areas to facilitate the analysis.

In-depth interviews were undertaken to collect detailed information on specific issues from five groups of purposively selected policy actors: policymakers, academia, researchers, representatives of civil society organization (CSO) and international development partner. A pre-tested in-depth interview guide was used to collect information from the respondents. The choice of respondents was informed by: a) the review of documents mentioned above, b) researchers’ knowledge of the actors’ involvement in the OHP development and c) initial meetings with the key stakeholders as part of consultations for selecting a policy within the case study. The list of respondents was continuously updated by the research team throughout the data collection process using the snowballing technique. The focus in selecting the respondents was to get a representation of the different groups of stakeholders who were involved in the policy process. Further informants were identified through a snowball technique. In all, nine respondents were interviewed: 3 policymakers, 3 academics, 1 researcher, 1 CSO representative and 1 development partner. Five respondents were part of the ten-man technical working group inaugurated in 2010 to develop the OHP [35]. Of these, two respondents had been involved in formulating two of the previous failed policy documents and their insights and criticisms of the current OHP were taken into consideration. Interestingly, seven of the nine respondents were primarily trained dental practitioners. All respondents had detailed knowledge of the policy processes for the OHP. Where key actors were not available for interviewing (e.g. due to retirement), their immediate colleagues possessing knowledge of the policy development were approached. Seven of the interviews were conducted face-to–face while two were through telephone interviews due to unavoidable logistic difficulties.

### Data analysis

Though little guidance exists for health policy analysis in LMICs, it is recommended that existing policy analysis tools can and should be used, as theories from health policy analysis in high-income countries can have resonance for health and developing countries [3]. The health policy triangle by Walt and Gilson was thus used to retrospectively analyze the OHP with regards to the context, process and actors [5]. The policy triangle offered a guide to systematically think about how these different factors may have affected the development of the OHP. This paper deliberately does not analyze the policy content; the fourth component of the policy triangle as it is covered in a different presentation. Interviews were recorded and transcribed verbatim. Data analysis was undertaken by use of the qualitative data analysis software (NVivo v10). This involved familiarization with the data, coding of data guided by a unified coding tree, indexing, charting, mapping and interpretation of data. The use of NVivo allowed for pre-determined themes to be explored and the emergence of new themes from the data. During the analysis, findings from interviews with different actor groups were continuously triangulated with results of document reviews. Triangulation between different methods was used to enhance the credibility of findings and to achieve a comprehensive picture of the OHP development in Nigeria.

### Ethical considerations

Ethical approval for the study was obtained from the Ethics Board of the University of Nigeria. The conventional ethical considerations for conducting research (preserving anonymity, ensuring confidentiality and obtaining informed consent) were complied with. At every stage written informed consent was obtained and respondents’ anonymity was protected.

## Results

The findings are presented in three different headings namely: context, policy process and actors.

### Context

This research explored internal and external contextual influences on development of the recently approved OHP in Nigeria. We adopt Dobrow *et al.’*s interpretation of internal context as the immediate environment in which the OHP is developed, and external context as wider political, disease-specific and extra-jurisdictional factors that shaped policy development and approval. Consistent with this, our research findings indicate that oral health was perceived as a neglected area of health care in Nigeria. According to some respondents, as there had been previous unsuccessful attempts to formulate an OHP due to failure to adhere to all policymaking stages in Nigeria, there was an overwhelming desire to follow through with a policy that will be nationally validated and accepted. As a result, this current OHP was formulated on a contextual background of: i) high level of need for oral healthcare; ii) low awareness of oral health amongst the Nigerian population; iii) perceived inadequacy of oral health services; and iv) inadequate and inequitable (urban/rural) distribution of human resources and financial needs for oral health.

Several other contextual factors at both the national and international levels influenced the entire process of policy development. A recurrent theme was the fact that findings of the NSHDP highlighted that there was no adopted OHP in Nigeria created a window of opportunity for developing an OHP that will be approved by the FEC. An alternative contextual opportunity that alluded to the principle of evidence-based practices was the view that the WHO had encouraged Nigeria, to produce and adopt an evidence-informed OHP. In line with this, the WHO provided actors with policy documents from other countries to aid the process. As suggested by the extract below, the desire to align with international standards and not be left out both at the national and international levels led to a buy-in by all stakeholders at various levels of the health care system:

*“[…] the availability of updated research findings, the sustained passion for the formulation of the policy, and the effective team of the various agencies; oral health related agencies in the ministry, including the Inter-country Centre for Oral Health, the regulatory bodies for the Dental therapists, the Dental technologists, dental nurses, meant they were all brought together as a team, with the deans of dental schools and the medical and dental council of Nigeria....we worked as a team, there wasn’t any rancour or any division in the course of the various sessions we had (Researcher)”.*

Whereas the preceding paragraphs and quote summarize the enabling factors; opportunities for developing the OHP and the consultative nature of the policy process, the principal threat to OHP development was captured by a quote:

*“Funding was the most important factor. If you want to bring some people together, somebody needed to provide the resources. That wasn’t forthcoming from the government’s side. So as partners, we had to support the process (development partner)”.*

See Table 1 below for other key enabling and constraining contextual factors on OHP development.

**Table 1:** **National and International Contextual Factors and their influences on development of OHP**

**Contextual factors**	**Influence on development of OHP in Nigeria**	
	**Enabling**	**Constraining**
**National**	● The National Strategic Health Development Plan (NSHDP) in progress.	● Lack of funding and government support especially for research activities.
	● Dedicated oral health research institute; Inter-country Centre for Oral health (ICOH)—a key source of generating and disseminating research evidence.	● Bureaucracy in the country which causes delay in approval and implementation of OHP.
		● Poor dissemination of research evidence from the ICOH to local levels which also leads to poor utilization
	● High level of interest and commitment of key stakeholders and a desire to align with international standards	
**International**	● International movement towards oral health policies and evidence-based practices.	● Inadequate financial support of the country towards evidence generation
	● Support of WHO and the World Bank with funding and dissemination of relevant policy documents.	

### Policy process

Although the development of the OHP was identified as a need in the Nigerian health sector, more than half of the respondents credited a Director of the Dentistry Division of the FMOH with spearheading the formulation of the OHP. Further probing revealed different respondents had different understandings of the policy process. One respondent mentioned how a road map for achieving objectives and developing a policy for improving oral health in Nigeria was agreed. He then went on to mention three other stages of policy development namely stakeholders meeting, policy formulation and policy approval. However, although they used different terms to describe stages of policy process, a synthesis of the perspectives of the nine respondents identified five stages of OHP policy process in Nigeria namely: a) agenda setting, b) problem identification, c) situation analysis, d) policy formulation, and e) policy approval. The launch of the policy document also appeared to be a prominent feature in the process. However, none of the respondents mentioned policy evaluation as a stage of policy process. The above stages are summarized by the following extracts:

*“Yes the agenda setting was…, you know I told you that we first of all made a proposal to the Ministry [of Health] and even after sending the proposal to the Ministry and it was approved, you know and there was a technical working group that was set up….(Policymaker)”.*

And further explained another policymaker:

*“From bottom-up every of the relevant stakeholders were identified and they were involved from the very beginning… there was a workshop that was organised to familiarise stakeholders with the draft policy before it was presented to the top management committee of FMOH and the Minister presented it as a memorandum to the National Council on Health and it was approved there. The Minister also presented it to the Federal Executive Council for approval and the President directed the Minister to present it to the National Economic Council for endorsement and all those processes were followed and that makes it a national policy (Policymaker)”.*

Apparently, the whole process from the need identification to the final adoption of the policy spanned two years (November 2010-November 2012). This was considered too long by some respondents who attributed the delay in adoption of the OHP to “bureaucracy and bottlenecks” within the government. According to a policy maker;

*“Like I told you, time, time, we didn’t think it will take us that long. It took us ehm a long time because of the bureaucratic bottlenecks. Before we could book appointments to see the ehm economic council, I don’t know how many months. Before we could book appointments to see the federal executive council, before there was another sitting of the national council of health, you know, those wasted a lot of time”.*

Though the process was perceived to be long, it is worth noting that these later stages (specifically waiting for approval by the Federal Executive Council and National Economic Council) were crucial for policy approval and adoption. As seen from a document review and supported by one respondent, this OHP was the first policy document to reach this final approval stage. As stated by an academic: “…*like I told you, ever since the 80s, there had been different oral health policies but we couldn’t refer to those as authentic because they were never approved at the right level so were really not implemented. And we really don’t want this one to suffer the same thing as earlier ones.”* Another respondent who is a policymaker noted that previous OHPs…“*did not follow due process*…” although he was vague about what the constraining factors were.

Document reviews revealed that three policy documents were drafted during the military era but were not followed through. However, the fourth policy document which had almost similar content as the present approved process, developed in the post-military democratic area was also not followed through. This new policy (approved in 2012) appears to be the only one which was submitted to the FEC, the highest policy making body in Nigeria, and approved. A policymaker noted that the buy-in of the current Minister of Health the catalyst for the policy process: *“…it is a bit different because the Minister actually was totally involved even though he wasn’t even the one coordinating the process, but was totally involved and got reports on regular basis and he also constituted the technical working group to fashion out a road map for oral health in Nigeria and which policy was actually a part”.*

Although the in-depth interview guide asked about the process that was followed in the formulation of the OHP, this may have been understood as the steps that stakeholders took amongst themselves in formulating the process as opposed to the conceptual stages of policy process in literature. Perhaps the way the question was phrased influenced their responses as reflected in the following accounts:

*“Well like I said there was a situational analysis in the country regarding oral health; what conditions constituted the biggest problem, what resources were available, and all other resources including human resources, and then what tools were available in terms of treatment guidelines… There was a group and the group did situational analysis and got to work. There were several workshops and meetings. And there were also several consultations. They got a list of key stakeholders to know who and who should be involved in the process. And they started meeting (development partner)”.*

*“…what happened was there was a stakeholders meeting and we got a lot of information from them, sat down together to brain storm on the information and you know we had sessions to think on…think through all the information and the feedback from them was what was utilized in the final writing (Academic)”*

In summary, the policy development process started with the identification of a need for formulating an OHP following the identification of the absence of a national OHP by the stakeholders of a National Strategic Health Development Plan (NSHDP). This was followed by a situational analysis which was conducted by a ten-man technical working group (TWG). This group further identified relevant stakeholders who had a series of meetings and consultations which led to the production of a draft policy document. The draft policy was passed through various stages of approval and adoption as seen above and was finally launched in November 2012. None of the respondents mentioned policy dissemination or evaluation as stages of policy process though one person alluded to the fact that the policy should have been in the implementation stage at the time of the interview, having been approved.

Table 2 summarizes the strengths and weaknesses of the OHP policy process from the perspectives of our respondents:

**Table 2:** **Strengths and Weaknesses of development of the OHP as perceived by respondents**

**Strengths of policy process**	**Weaknesses of policy process**
● Government support/political will	● Delayed approval by govt. of the agenda setting stage which delayed subsequent stages.
● Available research evidence	● Lack of funding
● Opportunity of a national Health Strategic Plan	● The time taken to generate and pull research evidence from different zones of the country.
● Diverse group of stakeholders	
● The leadership displayed by the OH secretariat	
● Broad based participation and commitment of stakeholders	
● Policy went through all the stages of approval in the national policy making process, despite delays.	

The OHP was formulated using existing evidence for policy which included research evidence generated mainly by ICOH and policy documents from other countries. It was however unclear whether the various strategies recommended in the policy document for improving oral health in Nigeria were based on evidence. Eight months after the launch of the OHP, very little had been done towards dissemination and implementation of the policy. One respondent remarked: “*The policy launch was one event at the national level, the policy documents had not been disseminated to the state and local government levels and as such the implementation had also not commenced*”. Another respondent (a policymaker) corroborated this by saying: “…*we should now be at the implementation stage…”* This delay was attributed to lack of funding towards these stages of the policy. However it was not apparent from the transcripts whether strategies for dissemination and implementation of the OHP were agreed upon during the policymaking process.

### Actors’ roles and relationships

Interviews show that the groups of actors involved in the OHP development process included: the academia/researcher, policymakers, development partners, CSO representatives and health workers. These groups comprised of individuals and/or organizations with different characteristics, e.g., the academics consisted of researchers and university teachers; policymakers consisted of executives in government parastatals and senior administrators in schools of dentistry; development partners were represented by the WHO; while CSOs consisted of professional bodies (Nigerian Dental Association) and regulatory bodies (Medical and Dental Council of Nigeria); and health workers’ group consisted of dental and para-dental health practitioners in private and public practice.

Our findings on actor types and characteristics also reveal that the key actors involved in the OHP process were the academia/researchers, development partners and policy makers. These groups of actors either had the mandate to make decisions or could influence policy through their funding power or ability to generate evidence in form of research findings. Most actors offered their technical expertise and the cordial relationship amongst them also contributed to facilitating the development of the policy. The ability of some actor groups (e.g. academics and WHO) to generate evidence or provide technical advice may explain their level of influence on policy process and involvement in developing the OHP in Nigeria.

One researcher commented:

“*We have the institute of oral health in Jos which is a WHO supported center. They do training, they do research, and they provide services as well. And even from that Centre we have done quite a lot of local research”.* Another respondent observed: *“if you want to know people really involved individually, you have dental surgeons, you have dental therapists these are essentially the researchers…they make the crux of the research data gathering team and we have a statistician in the Centre and data entry and ICT staff as well (Development partner)”.*

Regarding the centrality of role of WHO in the OHP process, some reported that the initial directive to produce an OHP was given to Nigeria by WHO and the organization followed through in the process. According to an academic:

*“I think the involvement of the WHO is quite important because that gives some kind of leverage to acceptance. You know there are too many areas in this country and too many groups actually struggling to get recognition. So I mean, if we really did not have the WHO approval and directive, may be oral health may have be lost in between other competing forces but I think with the WHO directive and with Nigeria being a major stakeholder in the WHO, I think that facilitated the approval for developing of Oral Health Policy in the country”.*

While roles played by researchers and development partners are clear, there was a difference in opinion amongst respondents about the representation of oral health professionals during policy development. For example, a policymaker felt that:

*“…the relevant professionals, how do I put it now the profession in dentistry were involved, yes were involved and then apart from that, Medical and Dental Council was involved, the Registrar was involved as part of the Technical Working Group and then in the finalization of the policy and the draft policy all the relevant stakeholders including NHIS, NPHCDA, the academia you know they were involved, the research institutions were involved.”,* while a health professional stated that *“the only thing that can be improved is to involve professionals more from time to time because they are the ones doing the work and they are the ones on ground and they are the ones in the field, so they will be able to know and say better of how or which direction they feel they can achieve the best. So I just feel they should improve on the involvement of the professionals”.*

The above difference of opinion may arise from variable involvement of actor groups in all deliberations during the policy process. This was alluded to by a respondent who stated that travelling round the country for different meetings sometimes posed logistic difficulties for actors attending meetings. This view was however contradicted by another respondent who suggested that stakeholders were kept updated. According to a Development partner,

*“Many more stakeholders would have been involved. Like I said, because of constraints such as funds and time, though the group was quite eager to complete the process it was quite difficult getting people to attend the meetings. People had … And you just can’t wait forever. We needed to go along. Some of those stakeholders are important, their inputs are important and perhaps would make some difference. But we made sure that even through factual means we got their inputs into the document. So they didn’t have to be present physically but we could send materials to you to make inputs. That also was a drawback”.*

It is also important to note that while lower level of participation of some actors may have affected their level of influence on policy process, yet, their participation may have enriched the process through their power of advocacy and lobbying. In particular advocacy groups such as the media were not involved in the evidence process of the OHP, and this may reflect the level of importance placed on the media in policy formulation, dissemination and implementation in Nigeria.

Amongst various recommendations made in relation to actors in the OHP was the need for timely involvement of more professionals, mothers, the youth and women groups, as well as local government officials at the grass root level in the process.

## Discussion and conclusions

Although several attempts were made at formulating an OHP in Nigeria in the past three decades, most of the attempts were unsuccessful. The first three attempts (between 1984 and 1999) at formulating the OHP failed because of an unfavorable political environment during military rule, whereas a fourth attempt at developing an OHP (2004-2009) failed due to non-observance of national stipulations for policy development. This demonstrates that no single factor can fully enable or constrain policymaking and that factors which may facilitate or constrain policymaking must always be contextualized in place and in time. According to Collins *et al*., “Policy formulation and implementation take place in a context which gives explanatory and historical meaning to that policy.*”* [36] The inference from this is that policies are expected to interact with the context in which they are developed and also produce some effect on it [37]. This was reflected in the Nigerian OHP process where many failed attempts during the military regime were subsequently approved and adopted a couple of years during a democratic regime.

Apart from the political environment in Nigeria, international influences also played a key role in shaping the OHP development. The findings highlight the influence of external policy actors such as WHO on the FMOH in shaping policy development and adoption of the OHP. This is akin to reports of external global influences (of e.g., the World Bank) on the policy agenda and formulation of child health and preventive health in LMICs [38].

The prevalent context also shaped the policy processes in the formulation of the OHP because whereas our findings show that the actors were well aware of some documented stages of policy process, there were however processes that were context specific and needed to be followed in the country; the absence of which had led to a failed attempt at formulating the OHP in the past. However, there are no definite boundaries between the stages as perceived by the policy actors and as outlined in literature. As noted by Foltz, some stages may be skipped, merged; while some stages may occur simultaneously [37,39,40]. Also, the findings of an incrementalist approach to policy development where it was perceived that dissemination of policy documents was a stage in the process of the OHP before implementation could occur has been commonly adopted in developing countries [5,37]. The noted delays and “bureaucratic bottlenecks” during the OHP development is not peculiar to this particular policy; it is the nature of policy making to be iterative and sometimes have very fuzzy boundaries between stages [5], but this can be limited or made worse by the interest and power of the actors involved. Other strengths and weaknesses in this study also resonate with a detailed analysis of the oral health care system in Nigeria by Adeniyi et al; and some of the strengths could be perceived as opportunities and some weaknesses as threats, were this paper to have used the SWOT (Strengths, weaknesses, opportunities and threats) analysis framework [24].

The characteristics and power of various actors although varied, were unified by a common interest of formulating a policy which will be adopted by the Federal Executive Council. It could almost be said that all the actors formed a single policy network driven by a common interest [41]. However, this is not always the case with all policy making, as was seen in Ghana’s experience during the formulation of the National Health Insurance Policy where stakeholders were primarily trying to protect the interests of their various constituencies [42]. Although the technocrats and bureaucrats were more in number than the donors, CSO and politicians [5], each group of actors played key roles at different stages of the policy process, and even when they were not playing key roles, remained involved to varying extents at all stages.

Whilst the policy makers, with support of high level government mandate and leadership provided ownership and legitimacy of the process, the academia /researchers provided scientific evidence for the policy and the WHO provided significant financial and technical support. As reported by Stover and Johnston in 1999, this pattern of synergy was noted in different African countries while formulating national HIV/AIDS policies [43]. The OHP process suffered a lot of delays and “bureaucratic bottlenecks” but was kept alive by the then Director of the Oral Health Division of the Federal Ministry of Health who could be considered throughout the process as the “champion”. The policy approval, being a political process, was where government support was overarching and particularly the Minister of Health who explicitly used his high level power and interest to facilitate the actualization of the OHP. This was the same situation during the formulation of the national HIV/AIDS policy in South Africa [43].

Lack of media representation and low level of representation of health workers (who would be directly responsible for implementation) was regretted by respondents in retrospect. However it was recommended that the media be involved during forthcoming implementation. The media is a strong policy network and has been known to influence various stages of the policy process, either in isolation or as a coalition [41,43].

A limitation of this study was the limited number of respondents, however using the snowball technique and triangulation ensured that important issues were not missed. Secondly we also ensured that we had reached saturation and hence felt comfortable to explore our questions with this sample size [44,45]. Though the policy triangle was used, the policy content was not analysed in this paper as it forms the content of another paper based on this study. In future, it would be pertinent to explore and analyze the implementation phase of the OHP.

In conclusion, the OHP was successfully formulated and approved through a complex inter-relationship of context, process and actors and clearly illustrates that none of these factors could have catalyzed the policy development in isolation. Availability of evidence is necessary but not sufficient. The wider social and political contexts in which actors develop policy can facilitate and/or constrain the actors’ roles and interests as well as the policy process. These must be taken into consideration at every stage of policy development in order to produce policies that will strengthen the health system. This is especially crucial in low and middle -income countries.

### Availability of supporting data

Study instruments and data are available on request from the corresponding author.

## Abbreviations

CSO: Civil Society Organisation
EU: European Union
EVAL-HEALTH: Developing and testing new methodologies to monitor and EVALuate HEALTH related EU-funded interventions in cooperation with partner countries
FEC: Federal Executive Council
HIV/AIDS: Human immunodeficiency virus/acquired immune deficiency syndrome
MDCN: Medical and Dental Council of Nigeria
NHIS: National health insurance scheme
NPHCDA: National primary health care development agency
NSHDP: National strategic health development plan
OHP: Oral health policy
WHO: World Health Organization
